# Integrating GWAS and transcriptomics to identify candidate genes conferring heat tolerance in rice

**DOI:** 10.3389/fpls.2022.1102938

**Published:** 2023-01-09

**Authors:** Pingping Li, Jing Jiang, Guogen Zhang, Siyu Miao, Jingbing Lu, Yukang Qian, Xiuqin Zhao, Wensheng Wang, Xianjin Qiu, Fan Zhang, Jianlong Xu

**Affiliations:** ^1^ Ministry of Agriculture and Rural Affairs (MARA) Key Laboratory of Sustainable Crop Production in the Middle Reaches of the Yangtze River (Co-construction by Ministry and Province), College of Agriculture, Yangtze University, Jingzhou, China; ^2^ Institute of Crop Sciences, Chinese Academy of Agricultural Sciences, Beijing, China; ^3^ College of Agronomy, Anhui Agricultural University, Hefei, China; ^4^ Shenzhen Branch, Guangdong Laboratory for Lingnan Modern Agriculture, Genome Analysis Laboratory of the Ministry of Agriculture, Agricultural Genomics Institute at Shenzhen, Chinese Academy of Agricultural Sciences, Shenzhen, China

**Keywords:** GWAS, transcriptome analysis, heat stress, candidate genes, germplasm resource, rice

## Abstract

**Introduction:**

Rice (*Oryza sativa L.*) production is being challenged by global warming. Identifying new loci and favorable alleles associated with heat tolerance is crucial to developing rice heat-tolerant varieties.

**Methods:**

We evaluated the heat tolerance at the seedling stage using 620 diverse rice accessions. A total of six loci associated with heat tolerance were identified by a genome-wide association study (GWAS) with ~2.8 million single nucleotide polymorphisms (SNPs).

**Results:**

Among the six detected loci, *qHT7* harbored the strongest association signal and the most associated SNPs. By comparing the transcriptomes of two representative accessions with contrasting heat tolerance, *LOC_Os07g48710* (*OsVQ30*) was selected as a promising candidate gene in *qHT7* due to the significant difference in its expression level between the two accessions. Haplotype 4 (Hap4) of *LOC_Os07g48710* was determined as the favorable haplotype for heat tolerance via the gene-based haplotype analysis. The heat-tolerant haplotype *LOC_Os07g48710*Hap4 is highly enriched in the tropical *Geng/Japonica* accessions, and its frequency has decreased significantly during the improvement process of rice varieties.

**Discussion:**

Based on the GWAS and transcriptomics integrated results, a hypothetical model modulated by *qHT7* in response to heat stress was proposed. Our results provide valuable candidate genes for improving rice heat tolerance through molecular breeding.

## Introduction

Rice (*Oryza sativa* L.) is one of the major food crops in the world. By 2050, global rice production will need to increase by 1.0%–1.2% annually to meet the growing food demand brought by population growth and economic development (Normile, 2008; Cramer et al., 2011). Unfortunately, heat stress has become a major limiting factor for rice growth and yield in recent years due to the rising global warming trend (Ahuja et al., 2010). Rice is sensitive to high temperature at almost all stages of growth and development. High-temperature stress can hasten the physiological maturity of rice, diminish assimilate accumulation, and cause permanent yield losses (Korres et al., 2017). Therefore, improving the heat tolerance of rice cultivars has become one of the major objectives of rice breeding worldwide.

High-temperature stress in rice induces an increase in reactive oxygen species (ROS), membrane damage, protein degradation, and a cascade of other heat stress reactions (Wahid et al., 2007; Bita and Gerats, 2013; Chen et al., 2021a). ROS can act as crucial signaling messengers in the early stages of heat stress. However, the ROS generated in the late stages of heat stress may cause damage to the cellular components of rice (Wahid et al., 2007; Zhang et al., 2019). For example, the fluidity of the plasma membrane increases during the early stages of heat stress, and cyclic nucleotide-gated channel proteins are responsible for signal transduction (Finka and Goloubinoff, 2014; Li et al., 2018). ROS, nitric oxide (NO), and Ca^2+^, which are second messengers that can trigger the expression of downstream genes and ROS-scavenging genes, contribute greatly to heat tolerance by controlling ROS concentrations in rice (Liu et al., 2006; Liu et al., 2008; Mittler et al., 2012; Zhu, 2016; Liu et al., 2020b; Chen et al., 2021a). The NAC transcription factors have been identified as vital regulators of stress responses. Under heat stress, the membrane-associated NAC gene *OsNTL3*, which directly binds to the *OsbZIP74* promoter and regulates its expression, may influence the level of H_2_O_2_ and malondialdehyde (MDA) and electrolyte leakage (Liu et al., 2020a). The heat stress-sensitive rice mutant *hts1* showed increased H_2_O_2_ accumulation, Ca^2+^ influx, as well as membrane and chloroplast damage in response to heat stress. In *hst1* mutants, the transcriptional activity of *HsfA2s* and its downstream target genes are repressed due to the disruption of heat signal transduction (Chen et al., 2021a). In *Arabidopsis thaliana*, the VQ (FxxxVQxLTG) motif-containing proteins interacting with WRKY transcription factors (TFs) may improve heat tolerance by regulating ROS production (Cheng et al., 2012; Cheng et al., 2021). Similarly, the functional module of *WRKY10*-*VQ8* plays a role in regulating thermotolerance by modulating the ROS balance in rice (Chen et al., 2022b).

As a complex trait in rice, heat tolerance is controlled by multiple genes and genetic networks. To date, at least 58 quantitative trait loci (QTLs) responsible for heat tolerance at different developmental stages have been identified in rice (Xu et al., 2021). Moreover, more than 23 genes involved in heat tolerance have also been cloned and functionally verified (Huang et al., 2022), leading to a better understanding of the genetic mechanisms underlying heat tolerance. Several studies have demonstrated that plant cells rapidly accumulate misfolded toxins when subjected to severe heat stress (Liu et al., 2020a). The proteasome degrades these toxic proteins more efficiently than they are reactivated by heat shock proteins (Zhang et al., 2019). *TT1*, which encodes the α2 subunit of the 26S proteasome, protects rice against heat stress by eliminating cytotoxic denatured proteins and balancing the heat response process (Li et al., 2015). *TT2*, encoding a Gγ subunit, confers heat tolerance in rice and is associated with wax retention at high temperatures (Kan et al., 2022). A major QTL *TT3*, consisting of two genes named *TT3.1* and *TT3.2*, enhances rice thermotolerance by transducing heat signals from the plasma membrane to the chloroplasts (Zhang et al., 2022). The tRNA 2-thiolation process is a highly conserved form of tRNA modification among organisms. Compared with *Geng* (*japonica*) rice, *Xian* (*indica*) rice exhibits higher heat tolerance, possibly due to a higher level of tRNA thiolation controlled by *SLG1*, which encodes the cytoplasmic tRNA2-thiolated protein 2 (Xu et al., 2020). As a tRNA^HIS^ guanylate transferase, *AET1* contributes to the modification of pre-tRNA^His^ and possibly regulates auxin signaling in rice to enable normal growth under high-temperature conditions (Chen et al., 2019).

Genome-wide association studies (GWAS), a powerful approach for identifying genotype-phenotype associations in natural populations, have been applied to dissect the genetic architecture of many complex traits in rice. Over the past decade, the loci underlying tens of rice traits were identified by GWAS, and several important genes were successfully verified by further transgenic experiments (Wang et al., 2020; Chen et al., 2022a). For heat tolerance, Wei et al. identified 77 loci associated with survival rate after heat treatment at the seedling stage by GWAS based on a panel of 255 rice accessions and identified *LOC_Os02g12890* as an important candidate gene that may respond to high-temperature stress based on integrated transcriptome analysis (Wei et al., 2021). In addition, Yang et al. detected ten heat-associated QTL by GWAS with 221 rice accessions and selected 11 promising candidate genes by combining GWAS and transcriptome data (Yang et al., 2022). However, the genetic basis of heat tolerance in rice remains unclear due to the small size and limited diversity of the previous panels used for GWAS.

In this study, we conducted a GWAS on heat tolerance at the rice seedling stage using 620 diverse accessions and compared the transcriptomes between heat-tolerant and heat-sensitive representative accessions. One potential candidate gene was identified at the major locus *qHT7* on chromosome 7, and the possible genetic pathways in response to heat stress were approached. This candidate gene could be employed for improving heat tolerance in future rice breeding. Our findings may also provide insight into the genetic mechanisms of heat stress response in rice.

## Materials and methods

### Plant materials and heat-stress treatment conditions

A panel of 620 rice accessions from the 3K Rice Genome Project (3K RG) (Wang et al., 2018) was used to evaluate heat tolerance at the seedling stage. The accessions contained 173 *Geng*, 411 *Xian*, 19 *admix*, 7 *Aus*, 9 *Basmati* and 1 unknown accessions (**Supplementary Table S1**). Twenty-four uniformly germinated seeds per replicate of each accession were sown in 96-well plates with holes at the bottom of each well. Then, the seeds were soaked in a container with tap water by placing the plates on scaffolds and were cultured in a phytotron at 28°C/25°C, 70% relative humidity and a 13-h light/11-h dark photoperiod. After 7 d, the seeds were transferred to Yoshida solution (pH 5.8-6.0), which was replaced every 3 d (Li et al., 2015). 13-day-old seedlings were exposed to 45°C for 3 d in a phytotron and then returned to normal conditions (28°C) for 7 d of recovery. The phytotron was set at 60% relative humidity and low light intensity (50–80 µM m^−2^ s^−1^) to minimize the influence of high light and hydrophobic stress (Hasanuzzaman et al., 2013). Then, the survival rate (SR) was calculated as the proportion of surviving seedlings. Based on the evaluation system (**Table 1** and **Supplementary Figure S1**), the leaf score of heat tolerance (SHT) was determined by visual inspection. At least three biological replicates were performed.

**Table 1:** The scale for leaf score of heat tolerance.

Score	Observation
1	The tip of the leaf is less than 1 cm
3	Tip drying extended up to ⅓ length in most leaves
5	⅓-⅔ of all leaves dried
7	More than ⅔ of all leaves fully dried
9	All seedlings apparently dead

### GWAS for heat tolerance

A total of 2,802,578 SNPs with a missing rate < 0.1 and minor allele frequency (MAF) ≥ 0.05 in the GWAS panel were filtered from the 3K-RG 4.8M SNP dataset (Alexandrov et al., 2014) by PLINK (Purcell et al., 2007). The GWAS based on a mixed linear model was performed with EMMAX (Kang et al., 2010) to identify the associations between SNPs and heat tolerance. The kinship matrix was calculated with an identical-by-state matrix using the pruned SNP subset (with the parameter “indep-pairwise 50 10 0.1” in PLINK) as a measure of relatedness between accessions. The eigenvectors of the kinship matrix were calculated using GCTA (with the parameter “-make-grm”) (Yang et al., 2011) and the first three principal components were used as covariates to control population structure. The effective number of SNPs (N) was calculated by the GEC software (Li et al., 2012), and a suggestive significance threshold of association (*P* = 2.29E-06) was determined by the Bonferroni correction method (1/N) for claiming significant SNPs. Manhattan plots of the GWAS results were plotted by the R package “qqman” (Turner, 2014). The significant SNPs within the 300-kb region were considered as a locus based on the previously reported linkage disequilibrium (LD) decay in 3K RG (Wang et al., 2018). The leading SNP within a locus was defined as the SNP with the lowest *P* value. Local LD block analysis was performed within 150 kb upstream and downstream of the leading SNP using the LDBlockShow (Dong et al., 2021).

### Haplotype analysis for candidate genes

The haplotype analysis was performed on each annotated gene in *qHT7* to identify candidate genes and unearth favorable haplotypes. The gene haplotypes were constructed with all SNPs in the coding sequence (CDS) and 1-kb promoter regions, respectively. The synonymous SNPs were merged into one haplotype following the method by Zhang et al. (Zhang et al., 2021). Duncan’s multiple range *post-hoc* tests were used to compare phenotypic differences between haplotypes (*n* ≥ 40 rice accessions). The module of Custom Genotyping and Comment (Rice) in MBKbase database (http://www.mbkbase.org/rice/customGT ) (Peng et al., 2019) was used to construct the candidate gene’s variety groups based on the SNP genotype of the published wild rice accessions and 3K RG with parameters “Sample Num: ≥ 40, ALT ≥ 5%, Missing ≤ 20%”. The haplotype network of a candidate gene was drawn by the minimum-spanning tree in Popart (Leigh and Bryant, 2015).

### Transcriptome analysis

One representative heat-tolerant *Xian* accession, FACAGRO 64 (F64), and a representative heat-sensitive *Xian* accession, PUILLIPINA KATARI (PK), were selected for transcriptome analysis. Shoot samples before and after 24 h of heat-stress treatment were collected and stored in liquid nitrogen, each with three biological replicates. Total RNA was extracted from shoot samples using the TRIzol reagent (Invitrogen) and then treated with RNase-free DNase I (Takara) to remove genomic DNA. Sequencing libraries were constructed according to the standard protocols provided by Illumina. The libraries were sequenced using Illumina NovaSeq 6000platform (150-bp paired ends) in Novogene (China). The raw sequence data reported have been deposited in the Genome Sequence Archive (Chen et al., 2021b) in National Genomics Data Center (CNCB-NGDC Members and Partners, 2022) with accession number CRA008760 that are publicly accessible at https://ngdc.cncb.ac.cn/gsa.

After removing adaptor and low-quality reads, clean reads were aligned to the Nipponbare reference genome (MSU v7.0) using HISAT2 (Kim et al., 2015). The gene expression levels based on fragments per kilobase of exon per million mapped fragments (FPKM) were calculated by StringTie (Pertea et al., 2015). Differentially expressed genes (DEGs) between two samples were identified with the DESeq2 package (Love et al., 2014) in R. The threshold for claiming DEGs was set as adjusted *P*-value (FDR) ≤ 0.05 and log fold change (FC) absolute value ≥ 1. Functional enrichment analysis of Gene Ontology (GO) and KEGG pathway was performed by clusterProfiler software (Yu et al., 2012). The threshold of adjusted *P*-value (FDR) < 0.05 was used to identify significantly enriched GO terms and KEGG pathways.

### Quantitative real-time PCR

Total RNA (1 ug) was reverse-transcribed into cDNA using FastKing gDNA Dispelling RT SuperMix kit (Tiangen; KR118-02). qRT-PCR analyses were performed with SuperReal PreMix Plus (SYBR Green) kit (Tiangen; FP205-2), including three biological replicates. *UBQ* was used as the internal control, and the relative expression levels of the target genes were quantified using the comparative cycle threshold (2^-ΔΔCT^) method (Livak and Schmittgen, 2001). Primers used for qRT-PCR are listed in **Supplementary Table S2**.

### Measurement of malondialdehyde content, ROS levels and enzyme activity

The shoots of F64 and PK under heat stress (45°C) for 72 h and control conditions were collected, respectively. The levels of superoxide dismutase (SOD), malondialdehyde (MDA), peroxidase (POD) and hydrogen peroxide (H_2_O_2_) in shoot tissue were determined using SOD, MDA, POD, H_2_O_2_ commercial kits following the manufacturer’s instructions (Suzhou Grace Biotechnology Co., Ltd.). Three biological replicates were included.

## Results

### Phenotypic variation in heat tolerance

**Figure 1:**
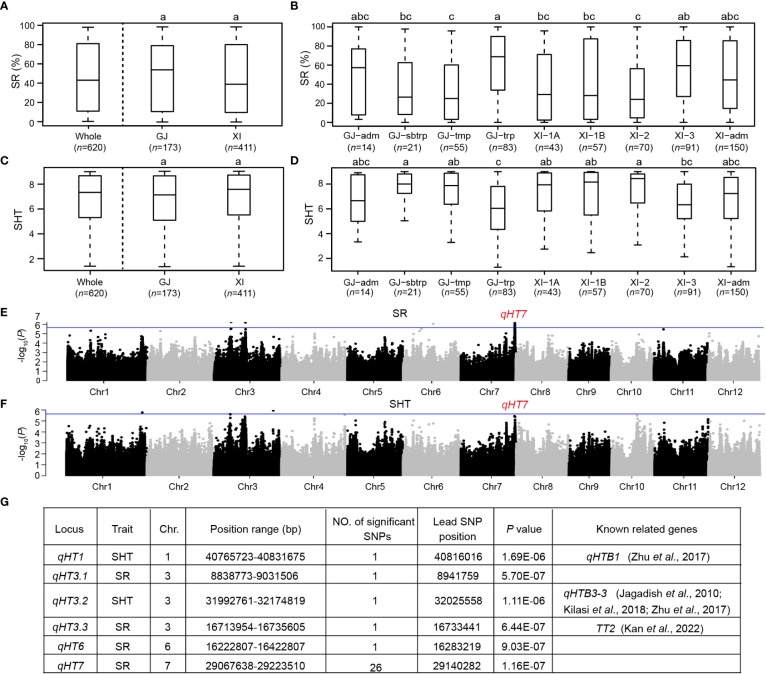
Phenotypic variations of heat tolerance and identification of loci associated with heat tolerance by GWAS in 620 rice accessions. **(A)** Box-plots of survival rate (SR) for the whole population, *Xian*/*Indica* (*XI*), and *Geng*/*Japonica* (*GJ*) subpopulations. **(B)** Box-plots of SR among *GJ*-*adm*, *GJ*-subtropical (*GJ-sbtrp*), *GJ*-temperate (*GJ-tmp*), *GJ*-tropical (*GJ-trp*), *XI-1A*, *XI-1B*, *XI-2*, *XI-3*, and *XI-adm* accessions. **(C)** Box-plots of leaf score of heat tolerance (SHT) for the whole population, *XI* and *GJ* subpopulations. **(D)** Box-plots of SHT among *GJ*-*adm*, *GJ-sbtrp*, *GJ-tmp*, *GJ-trp*, *XI-1A*, *XI-1B*, *XI-2*, *XI-3*, and *XI-adm* accessions. **(E)** Manhattan plots of GWAS results for SR. **(F)** Manhattan plots of GWAS results for SHT. **(G)** Genome-wide significant loci for SR and SHT. In A-D, different letters indicate significant differences (*P* < 0.05, Duncan’s multiple range *post-hoc* test). In E, F, the horizontal blue lines represent the suggestive significant threshold (*P* = 2.29E-6).

The phenotypic measurements of SR and SHT showed a considerable variation in heat tolerance at the seedling stage among the 620 rice accessions (**Figure 1** and **Supplementary Table S1**). The mean SR in the whole population was 46.9%, ranging from 0 to 100.0%. Similarly, the mean SHT was 6.82, with a range of 1.29 to 9.00. Heat tolerance at the seedling stage did not vary significantly between *Xian* and *Geng* subpopulations (**Figures 1A, C**). Among the four *Geng* subgroups, most accessions with high heat tolerance belonged to *GJ-trp* (**Figures 1B, D**). Within *Xian* subpopulation, the average heat tolerance of *XI-3* accessions was higher than those of other *Xian* subgroups. The heat tolerance of the *GJ-trp* accessions was similar to that of the *XI-3* subgroups. Moreover, most *GJ-trp* and *XI-3* accessions are both from Southeast Asia islands.

### GWAS for heat tolerance

Thirty-one SNPs significantly associated with heat tolerance were identified in the 620 accessions, including 29 and 2 SNPs associated with SR and SHT, respectively (**Figures 1E, F** and **Supplementary Table S3**). Among the significant SNPs, 6, 3 and 22 were located in the promoter, CDS and intergenic regions of 17 annotated genes, respectively. Based on the local LD block analysis, we combined these significant SNPs into six loci distributed on rice chromosomes 1, 3, 6, and 7 (**Figure 1G**). By comparing the 58 previously reported QTLs for heat tolerance (Xu et al., 2021) and 23 known genes involved in heat tolerance (Huang et al., 2022), three genes/QTLs, *qHTB1* (Zhu et al., 2017), *qHTB3-3* (Jagadish et al., 2010; Zhu et al., 2017; Kilasi et al., 2018), and *TT2* (Kan et al., 2022) were also found in the region of *qHT1*, *qHT3.2*, and *qHT3.3*, respectively. Out of the six loci, *qHT7* (Chr7: 29067638-29223510 bp) was determined as the major locus since it contained the most and strongest association signals (**Figure 1G**).

### Physiological comparison of two rice accessions with different levels of heat tolerance in response to high temperature

**Figure 2:**
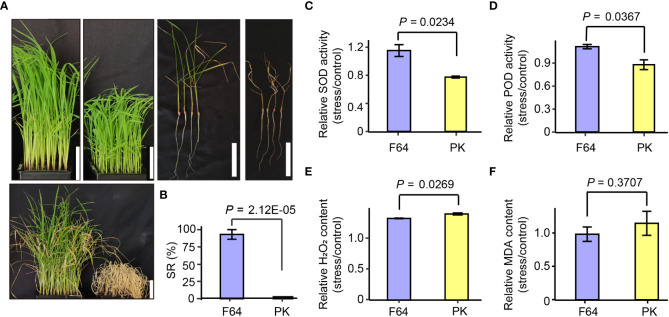
The differences in morphological and physiological performances between two rice accessions differing in their tolerance to heat stress. **(A)** Growth images of the heat-tolerant accession FACAGRO 64 (F64) and the heat-sensitive accession PUILLIPINA KATARI (PK) before and after heat stress treatment. Scale bars = 5 cm. **(B)** SR of the two rice accessions in 7 d after heat stress. Data are extracted from **Supplementary Table S1**. **(C–F)** The relative SOD, POD, H_2_O_2_ and MDA content between heat stress for 72 h and control conditions. Data shown in the form mean ± standard deviation of three biological replicates. The significant difference between the two groups was calculated using two-tailed Student’s *t*-test.

F64 was highly tolerant to heat stress, with an average 95.8% ± 7.2% SR, which only slightly dried at the tips after 7 d of recovery from heat stress (**Figures 2A, B**). PK was extremely sensitive to heat stress, with an average SR of 0% ± 0%, and all of the seedlings were apparently dead. To examine the cell membrane damage and redox homeostasis caused by heat stress in the two rice accessions, we compared the physiological traits between F64 and PK under heat stress for 72 h (**Figures 2C–F**). Although there was no statistically significant difference in the relative MDA content between the two accessions (**Figure 2F**), the relative H_2_O_2_ content of F64 after 72 h of heat stress was significantly lower than that of PK (**Figure 2E**). Moreover, the relative activity of the antioxidant enzymes SOD and POD were significantly higher in F64 than in PK (**Figures 2C, D**). These results suggested that F64 suffered less damage to cell membranes under heat stress than PK, possibly due to more effective active detoxification by ROS scavenging regulation in F64.

### Comparative transcriptome profiling between two rice accessions differing in their heat tolerance

**Figure 3:**
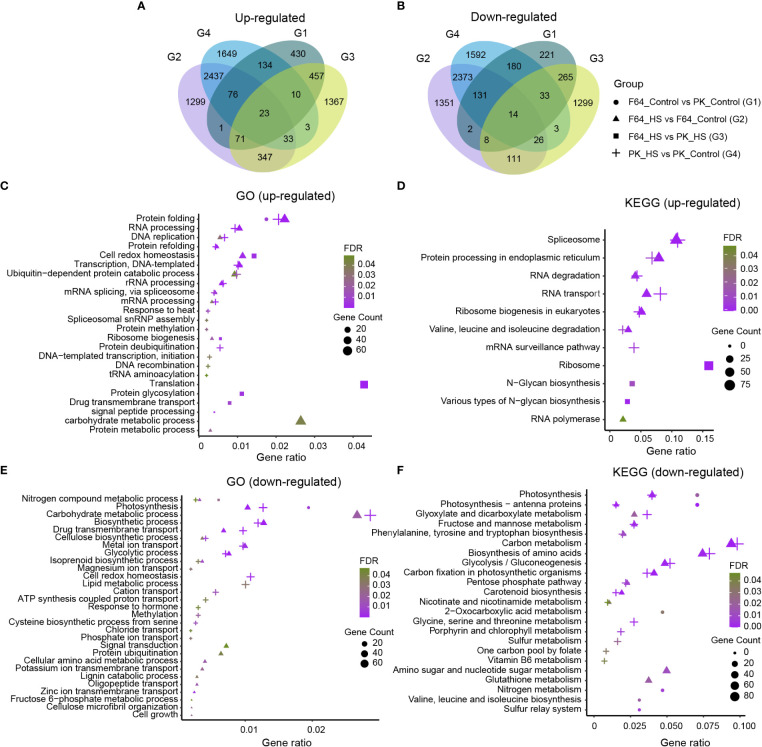
The transcriptome analysis of two rice accessions differing in their tolerance to heat stress (HS). **(A)** Venn diagrams showed the up-regulated differentially expressed genes (DEGs). **(B)** Venn diagrams showed the down-regulated DEGs. **(C, D)** GO and KEGG enrichment analysis of up-regulated DEGs. **(E, F)** GO and KEGG enrichment analysis of down-regulated DEGs. In c, e, only biological process GO terms were shown. G1: F64_Control vs PK_Control; G2: F64_HS vs F64_Control; G3: F64_HS vs PK_HS; G4: PK_HS vs PK_Control.

In order to reveal the differences in transcriptome response to heat stress at the seedling stage between rice accessions with different levels of heat tolerance, we compared F64 (a representative heat-tolerant accession) with PK (a representative heat-sensitive accession) using RNA-seq analysis (**Supplementary Table S4**). A total of 2056, 8303, 4070 and 8717 DEGs were identified for G1 (F64 vs PK under control conditions), G2 (heat stress vs control in F64), G3 (F64 vs PK under heat stress) and G4 (heat stress vs control in PK), respectively. Among them, 1202, 4287, 2311 and 4365 DEGs were upregulated, and 854, 4016, 1759, and 4352 DEGs were down-regulated in G1, G2, G3 and G4, respectively (**Figures 3A, B**). A series of biological processes and pathways involved in response to heat stress were commonly identified in both heat-tolerant and heat-sensitive accessions. Specifically, gene ontology (GO) analysis for the G2 and G4 DEGs, which were significantly regulated by high temperature in heat-tolerant and heat-sensitive accessions, respectively, showed that the common biological processes were mainly upregulated in protein folding (GO: 0006457) and RNA processing (GO: 0006396) (**Figure 3C**), and were primarily downregulated in carbohydrate metabolic process (GO: 0005975), biosynthetic process (GO: 0009058), metal ion transport (GO: 0030001), and glycolytic process (GO: 0006096) (**Figure 3E**). Similarly, six KEGG pathways, including spliceosome (map03040), protein processing in endoplasmic reticulum (map04141), RNA degradation (map03018), RNA transport (map03013), ribosome biogenesis in eukaryotes (map03008), and valine, leucine and isoleucine degradation (map00280), were significantly enriched both in the G2 and G4 upregulated DEGs (**Figure 3D**). For the G2 and G4 downregulated DEGs, 12 common KEGG pathways were significantly enriched, such as carbon metabolism (map01200), biosynthesis of amino acids (map01230), etc (**Figure 3F**). The results suggest that the aforementioned biological processes and pathways mentioned above should be the components of regulatory mechanisms underlying heat tolerance in rice.

Moreover, several unique GO terms and KEGG pathways were identified in G2 DEGs compared to the G4 DEGs. Four specific GO terms and one KEGG pathway were significantly enriched in G2 upregulated DEGs compared to G4 upregulated DEGs, including cell redox homeostasis (GO: 0045454), ribosome biogenesis (GO: 0042254), carbohydrate metabolic process (GO: 0005975), protein metabolic process (GO: 0019538), and RNA polymerase (map03020). In contrast, the divergence between G2 and G4 downregulated DEGs was much greater according to the number of unique GO terms and KEGG pathways (**Figures 3E, F**). Interestingly, cell redox homeostasis (GO: 0045454) was specifically enriched in G4 downregulated DEGs compared to G2 downregulated DEGs.

Genes involved in cell redox homeostasis are usually triggered in plants tolerant to abiotic stresses (Awasthi et al., 2015). Given that the genes related to cell redox homeostasis exhibited contrasting responses to heat stress in the two rice accessions with different heat tolerance, we further compared the expression profiles of 62 DEGs related to cell redox homeostasis between F64 and PK under control and heat stress conditions (**Supplementary Figure S2**). There were 34 (55%) common, 12 (19%) F64-specific and 10 (16%) PK-specific DEGs regulated by heat stress in the two accessions. The results suggest that cell redox homeostasis should play an important role in rice heat tolerance.

### Integrating GWAS and RNA-seq to identify candidate genes for *qHT7*

**Figure 4:**
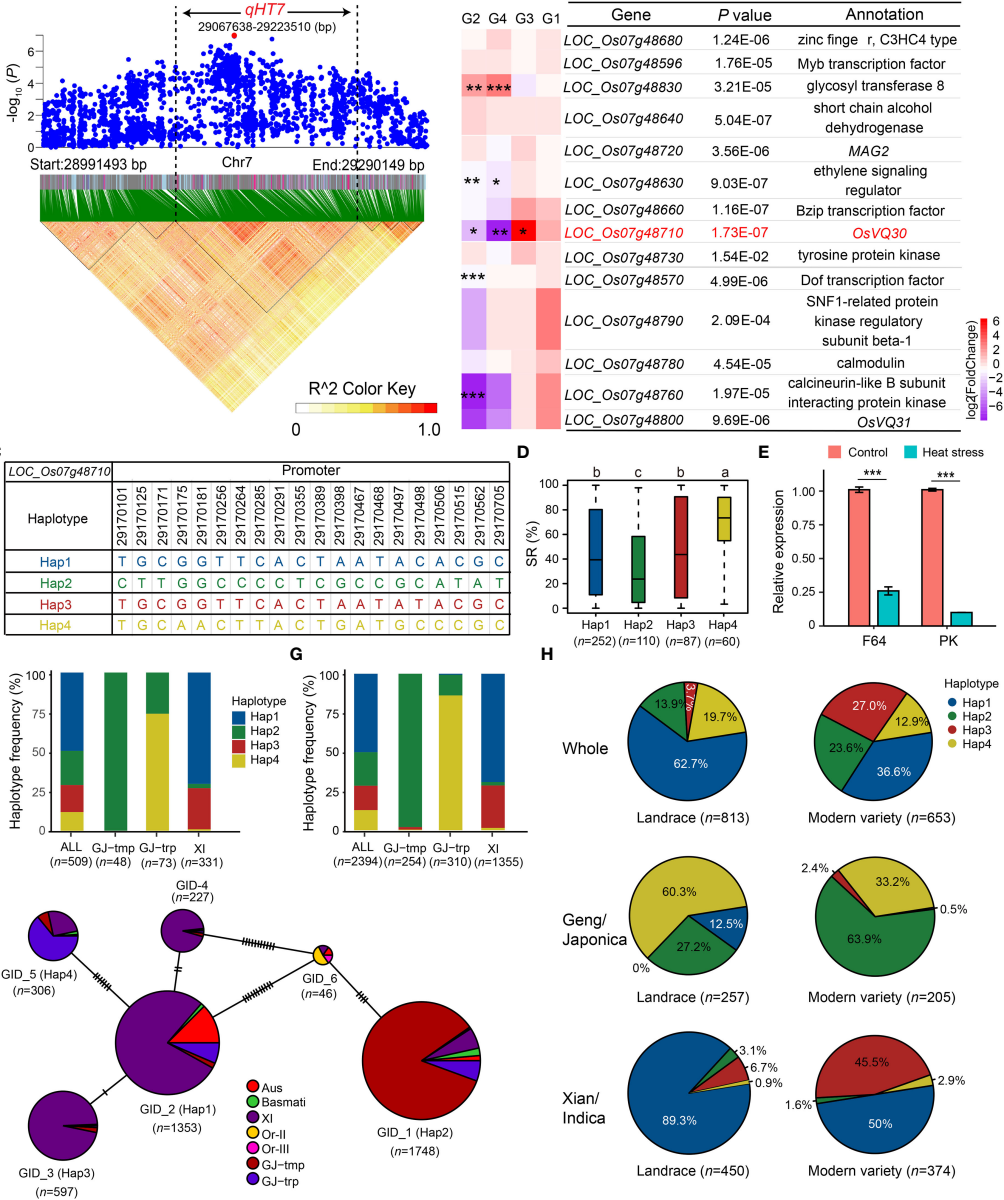
Candidate gene analysis of *qHT7*. **(A)** Local Manhattan plot (top) and LD analysis (bottom) of 150-kb upstream and downstream around the lead SNP rs7_29140282. The red dot is the lead SNP, and its LD block region is marked by the black dotted lines. **(B)** Relative expression of 14 annotated genes in *qHT7*. *** FDR < 0.001, ** FDR < 0.01, * FDR < 0.05. G1: F64_Control vs PK_Control; G2: F64_HS vs F64_Control; G3: F64_HS vs PK_HS; G4: PK_HS vs PK_Control. **(C)** Haplotype of *LOC_Os07g48710*, which is the promising candidate gene of *qHT7*. **(D)** The distribution of SR in the whole population for the four major haplotypes (*n* > 40 accessions) of *LOC_Os07g48710*. Different letters above each boxplot indicate significant differences among haplotypes (*P* < 0.05, Duncan’s multiple range *post-hoc* test). **(E)** Verification of the relative expression of *LOC_Os07g48710* in F64 and PK under heat stress 24 h by qRT-PCR. *UBQ* was used as an internal control. The figure presents the relative expression levels of *LOC_Os07g48710* relative to that under control conditions in each accession. Bars represent standard deviation of three biological replicates. ***P < 0.001 (two-tailed Student’s *t*-test). **(F, G)** Frequency of the four major haplotypes of *LOC_Os07g48710* in the GWAS panel **(F)** and in 3K RG **(G)**. **(H)** Haplotype frequency distribution of *LOC_Os07g48710* in landrace and modern variety of 3K RG. The type of each accession was from the metadata of 3K RG (Wang et al., 2018). **(I)** Haplotype network of *LOC_Os07g48710* retrieved from MBKbase (Peng et al., 2019) (http://www.mbkbase.org/rice/, query date: October 25^th^, 2022). Circle size of a given haplotype is proportioned to its number of accessions. Letter *n* indicates the number of rice accessions belonging to the corresponding haplotype in **D** and **I**, subpopulation in **F** and **G**, or variety type in **H**, respectively.

Based on the Nipponbare reference genome IRGSP 1.0, 28 genes were annotated in *qHT7* (**Figure 4A** and **Supplementary Table S5**). Candidate genes for heat tolerance were selected based on the following criteria: (1) functionally related to abiotic stresses based on the annotation of Nipponbare reference genome, GO annotation, and literature search; (2) significant differences in heat tolerance among gene haplotypes. Consequently, 14 candidate genes were identified (**Figure 4B** and **Supplementary Table S5**). To further screen the promising candidate genes, we examined the expression profiles of the 14 candidate genes using the transcriptomic datasets of F64 and PK. As a result, five DEGs (*LOC_Os07g48830*, *LOC_Os07g48630*, *LOC_Os07g48710*, *LOC_Os07g48570*, and *LOC_Os07g48760*) were selected (**Figure 4B**). We also verified the expression of the five genes by qRT-PCR (**Figure 4E** and **Supplementary Figure S3**). Among the five genes, only the expression level of *LOC_Os07g48710* was significantly higher in the heat-tolerant accession F64 than that in the heat-sensitive accession PK under heat stress (**Figure 4E**), which was consistent between the RNA-seq and qRT-PCR results. Thus, *LOC_Os07g48710*, encoding a VQ domain-containing protein, was determined as a promising candidate gene for the further analysis.

Mining heat-tolerant allele is helpful in improving the heat tolerance of rice through molecular breeding. To examine the favorable haplotype of the promising candidate gene of *qHT7*, *LOC_Os07g48710*, we performed the haplotype analysis using CDS and 1-kb promoter SNPs in 3K RG. Due to no SNPs detected in the CDS of *LOC_Os07g48710*, we identified four major haplotypes (*n* ≥ 40 accessions) using 20 SNPs (MAF ≥ 0.05 and heterozygous rate < 0.05) in its 1-kb promoter region in the GWAS panel (**Figure 4C**). Among the four haplotypes, Hap4 with significantly higher SR was determined as the favorable haplotype (**Figure 4D**), which was significantly enriched (*P* = 2.63E-54) in *GJ-trp* accessions of the GWAS panel (**Figure 4F**). For the 3K RG, the heat-tolerant haplotype *LOC_Os07g48710* ^Hap4^ was also highly enriched in the *GJ-trp* accessions (*P* = 1.60E-287) (**Figure 4G**). In contrast, *LOC_Os07g48710* ^Hap4^ was virtually absent in *GJ-tmp* subpopulation and *Xian* subpopulation (**Figures 4F, G**). To explore the origin and spread of Hap4, the haplotype network of *LOC_Os07g48710* was analyzed, showing that Hap4 possibly evolved from Hap1 (**Figure 4I**). Furthermore, the proportion of *Geng* accessions with *LOC_Os07g48710* ^Hap4^ dropped dramatically from 60.3% in landrace to 33.2% in modern variety (**Figure 4H**).

## Discussion

Understanding the genetic mechanisms underlying heat tolerance is vital to developing heat-tolerant rice varieties to adapt to global warming. In this study, different rice subpopulations exhibited different responses to heat stress at the seedling stage. Most *GJ-trp* and *XI-3* accessions, mainly from Southeast Asia islands, showed more tolerant to heat stress than other accessions, suggesting that the high temperature of the tropical environment may be the driving force in the evolution and breeding selection of heat tolerance in rice.

Heat tolerance is a quantitative trait controlled by a complex genetic network in rice. Fortunately, integrating GWAS and transcriptome analysis is now available as a powerful method for identifying candidate genes associated with complex traits. In this study, six loci associated with heat tolerance at the seedling stage were identified by GWAS. By comparing the previously reported cloned genes for heat tolerance with the GWAS results, *TT2*, a well-known heat-tolerant QTL (Kan et al., 2022), was co-localized with *qHT3.3*. Loss-of-function *TT2* allele has been found to exhibit increased thermotolerance and wax retention at high temperatures. In addition, we identified two loci, *qHT1* and *qHT3.2*, which were co-localized with previously reported QTL for heat tolerance, *qHTB1* and *qHTB3-3*, respectively (Jagadish et al., 2010; Zhu et al., 2017; Kilasi et al., 2018).

Notably, a novel major locus *qHT7* (Chr7: 29067638-29223510 bp) associated with heat tolerance at rice seedling stage was identified, and a promising candidate gene (*LOC_Os07g48710*) was predicted. The coding sequence of *LOC_Os07g48710* is highly conserved in the 3K RG with only one major gene-CDS-haplotype (Zhang et al., 2021). In contrast, at least four major haplotypes based on the natural variations in the promoter region exist in rice germplasm (**Figure 4C**). Moreover, although the expression levels of *LOC_Os07g48710* were both inhibited in heat-tolerant accession F64 (with the favorable haplotype *LOC_Os07g48710* ^Hap4^) and heat-sensitive accession PK (with the non-favorable haplotype *LOC_Os07g48710* ^Hap2^) under heat stress, the expression level of *LOC_Os07g48710* was significantly higher in F64 than in PK, implying natural variations in its promoter region are likely to be causal SNPs responsible for heat tolerance. The heat-tolerant haplotype *LOC_Os07g48710* ^Hap4^ is subpopulation-specific, which is preferentially carried by *GJ-trp* accessions rather than *GJ-tmp* and *Xian* accessions (**Figures 4F, G**), suggesting that *qHT7* may partially explain the phenotypic variation of heat tolerance in rice germplasm. Thus, the favorable haplotype, *LOC_Os07g48710* ^Hap4^, may serve as a potential alternative for improving the heat tolerance of rice varieties by gene editing or marker-assisted selection. Further experiments should be conducted to validate the function of *LOC_Os07g48710* on heat tolerance and evaluate the breeding value of its favorable haplotype in developing new rice varieties with enhanced tolerance to heat stress.

**Figure 5:**
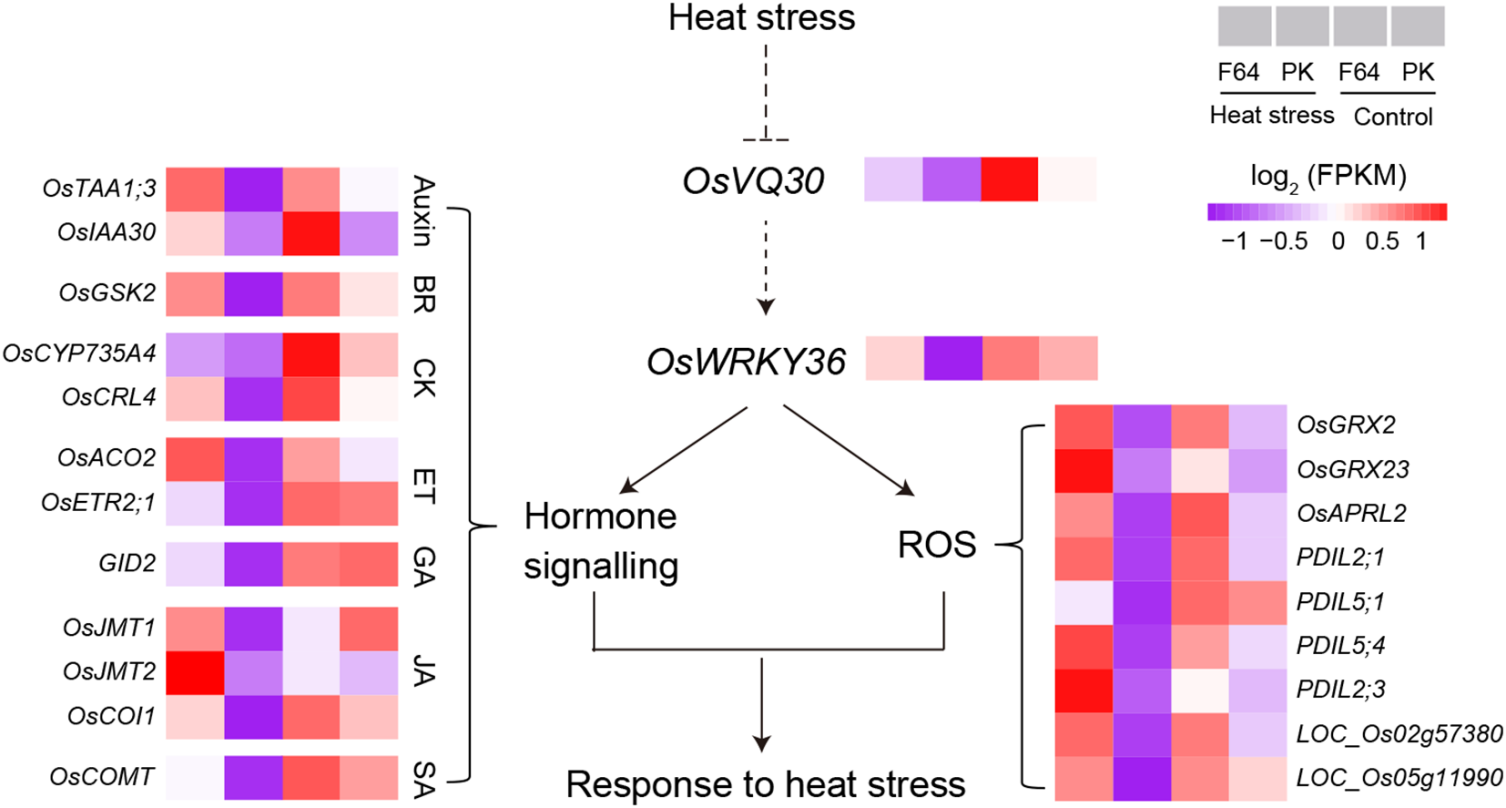
Hypothetical model of *qHT7* responses to high-temperature stress in rice. The model is proposed based on the genome and transcriptome analysis in this study. A complete list of genes is shown in **Supplementary Table S6**.

The VQ proteins are plant-specific transcriptional regulatory factors that can fine‐tune the regulatory pathway in response to abiotic stresses *via* interacting with TFs (Kim et al., 2013; Jing and Lin, 2015). The rice genome contains at least 39 VQ genes (numbered *OsVQ1* to *OsVQ39*), in which *LOC_Os07g48710* (*OsVQ30*) can be induced by drought stress rather than ABA treatment (Kim et al., 2013). Different VQ proteins can bind to the WRKY DNA-binding domain to modulate the expression of downstream genes and phytohormone signaling pathways in response to high-temperature stress (Cheng et al., 2012; Wang et al., 2015; Zhou et al., 2016; Jiang et al., 2017; Jiang et al., 2018; Cheng et al., 2021; Chen et al., 2022b). Cheng et al. (Cheng et al., 2021) have reviewed the WRKY-VQ protein interaction regulatory mechanism that regulates plant growth under high-temperature stress. For example, *WRKY39* activates SA- and JA-activated signaling pathways that promote the response to heat stress (Li et al., 2010). The functional module of WRKY10-VQ8 regulates heat tolerance by modulating the ROS balance in rice (Chen et al., 2022b). In this study, we identified 22 G3-DEGs with a similar expression pattern as *LOC_Os07g48710*, including a *WRKY* gene (*OsWRKY36*), 12 genes related to hormone biosynthesis/signaling, and nine genes related to cell redox homeostasis (**Supplementary Table S6**). Based on these genes that are likely connected to *LOC_Os07g48710* (*OsVQ30*), we hypothesize the putative regulatory model mediated by *qHT7* in response to heat stress in rice (**Figure 5**). In this model, heat stress strongly inhibits the expression of *OsVQ30* and *OsWRKY36*, and the WRKY-VQ module may regulate their target gene expression to respond to high-temperature stress in rice. Further studies are required to verify the hypothesis.

## Data availability statement

The datasets presented in this study can be found in online repositories. The names of the repository/repositories and accession number(s) can be found below: https://ngdc.cncb.ac.cn/gsa , CRA008760.

## Author contributions

JX, FZ and XQ designed the experiment; PL, JJ, GZ, SM, JL and YQ performed all the phenotypic evaluation; PL, FZ, XZ and WW performed analysis and interpretation of the data; PL and FZ drafted the manuscript; FZ and JX revised the MS. All authors contributed to the article and approved the submitted version.
