# Supplementary material for: Integrating GWAS and transcriptomics to identify candidate genes conferring heat tolerance in rice
Source: Front Plant Sci. 2023 Jan 9;13:1102938. doi: 10.3389/fpls.2022.1102938 (PMC9868562; doi:10.3389/fpls.2022.1102938)
Supplement: Supplementary file 1 [file DataSheet_1.pdf]

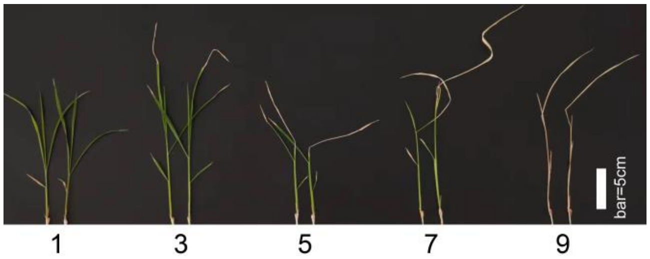

Supplementary Fig. S1. Morphological responses of five rice accessions based on the leaf score of heat tolerance (SHT) via visual assessment shown in Table 1.

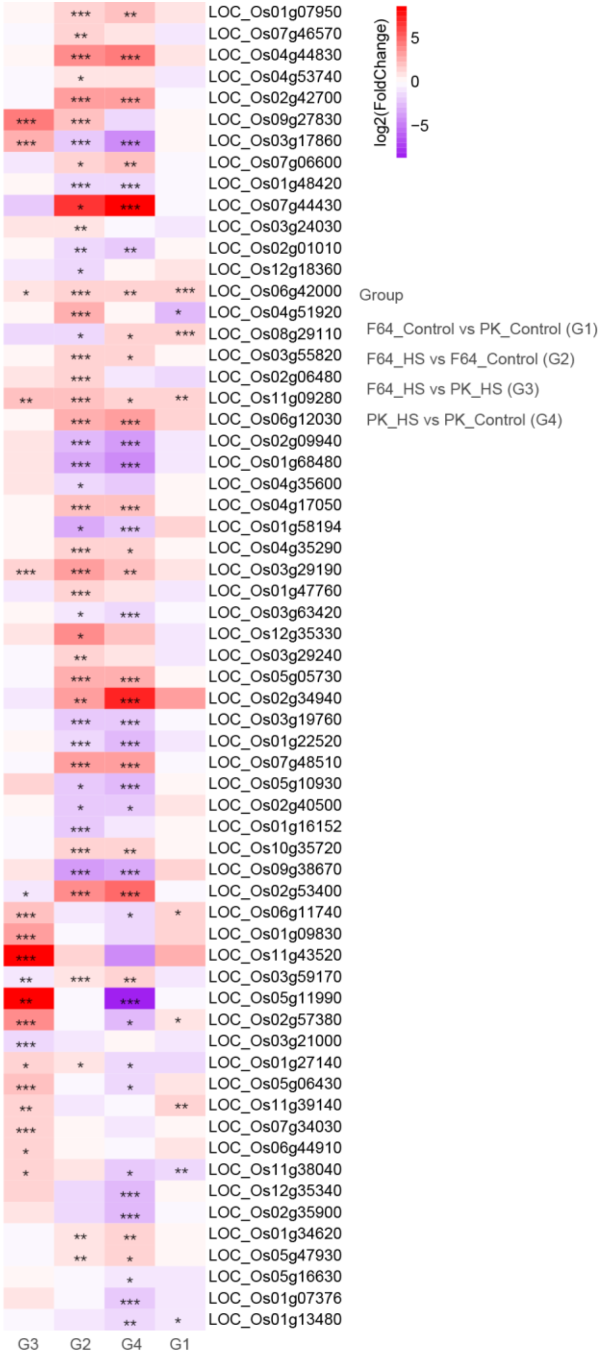

Supplementary Fig. S2. Gene expression patterns of 62 genes involved in cell redox homeostasis in F64 and PK under heat stress (HS) and control conditions.

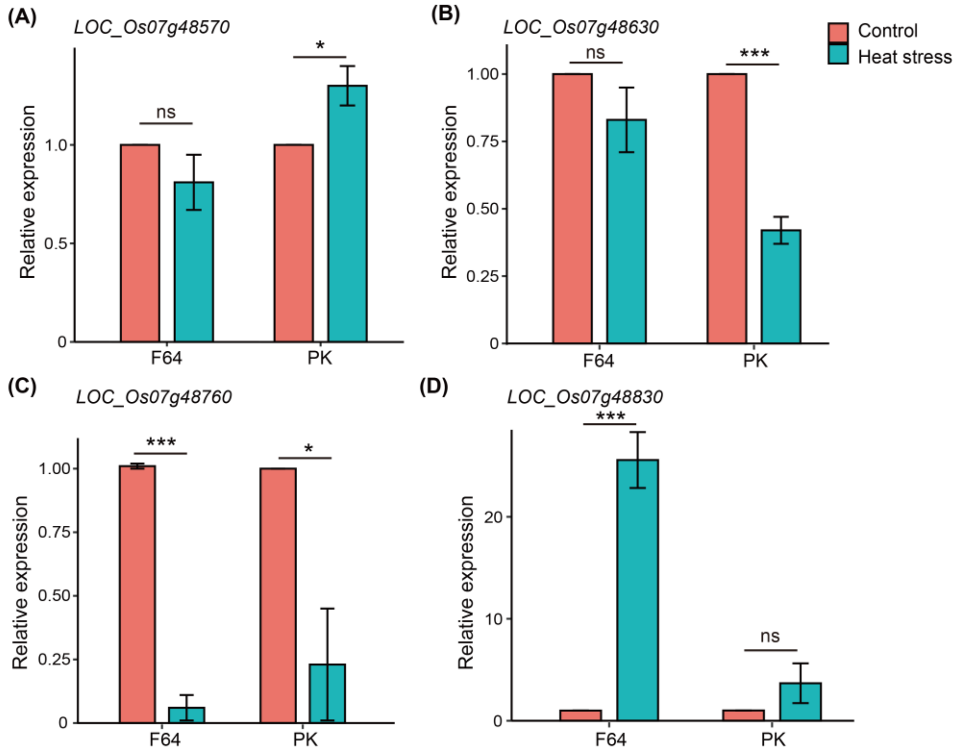

Supplementary Fig. S3. qRT-PCR results of other four *qHT7* candidate genes in F64 and PK under heat stress 24 h and control conditions.
